# 5-Year data analysis of patients following abdominal wall endometrioma surgery

**DOI:** 10.1186/s12905-014-0151-4

**Published:** 2014-12-05

**Authors:** Tahere Khamechian, Javad Alizargar, Tahere Mazoochi

**Affiliations:** Anatomical Sciences Research Center, Kashan University of Medical Sciences, Kashan, I.R Iran; Student Research Committee, Kashan University of Medical Sciences, Kashan, I.R. Iran; Gametogenesis Research Center, Kashan University of Medical Sciences, Kashan, I.R Iran

**Keywords:** Endometrioma, Abdominal wall endometriosis, Scar endometriosis

## Abstract

**Background:**

Endometriosis is a disorder in which an ectopic endometrial tissue grows outside the uterine cavity. The ectopic endometrium embedded in the subcutaneous fatty layer and the muscles of abdominal wall is called as abdominal wall endometriosis (AWE). AWE is a rare condition; however, it is usually known to develop along with previous surgical scars. Caesarean section and hysterectomy are considered to be commonly associated with the development of AWE.

**Methods:**

We evaluated the data of the patients who underwent AWE surgery between March 2009 and March 2014.

**Results:**

The mean age of the patients was 32.5 years. We found a previous history of caesarean section in all of the patients. The most frequent symptoms of the patients were abdominal mass sensation and abdominal pain. Invasion of endometriosis to fat layer, fascia, muscular layer, and peritoneum was recorded. Three masses were located within the scar regions.

**Conclusions:**

We can conclude that there is a high prevalence of caesarean sections among the women with AWE.

## Background

Endometriosis was first described by an Austrian pathologist, Karl Freiherr von Rokitansky in 1860 who referred to the disease as adenomyoma [1]. Endometriosis is a rare condition in which ectopic endometrial tissue grows outside the uterine cavity and responds to hormonal stimuli [2]. Although its prevalence in adult women is not completely known, it is said to occur in 5%–10% [3]. Although it is more common in pelvic regions such as the ovaries, posterior cul-de-sac, ligaments of the uterus, pelvic peritoneum, and rectovaginal septum [4], it can also be extrapelvic. The extrapelvic occurrence of endometriosis is rare and it occurs more commonly in abdominal wall followed by umbilicus, vulva, appendix, ileum, hernia sac, and colon [5]. It is known to develop spontaneously [6] and is usually found near or at the site of scars of surgeries like hysterectomies, caesarean sections, laparoscopies, tubal ligations, and episiotomies [4,7]. These sites propose the “iatrogenic direct implantation theory”, which emphasizes on the implantation of the escaped endometrial cells caused by the surgery into the surgical wound [8].

The ectopic endometrium is embedded in subcutaneous fatty layer and muscles of abdominal wall in abdominal wall endometriosis (AWE) [9]. AWE is considered very rare with the overall prevalence of around 0.01–1% after caesarean section [10]. In addition, AWE is often mistaken with some surgical conditions like abscess, lipoma or incisional hernia [11]. Although manifestations of endometriosis may range from dysmenorrhea to even bowel perforation [12], the most prevalent clinical feature of endometriosis includes a painful mass with a cyclic pain that exacerbates with menstruation [4].

The present study presents the features of AWE in a five-year experience of surgeons from Shahid Beheshti Hospital. It describes the patients’ characteristics and manifestations of the disease.

## Methods

After obtaining written informed consent, the data of the patients who underwent surgery for AWE between March 2009 and March 2014; including age, parity, related past medical history, chief complaint, the exact site of AWE, and any information gained after the surgery were entered in the study. This study has complete approval by the institutional review board of Kashan University of Medical Sciences.

## Results

**Figure 1:**
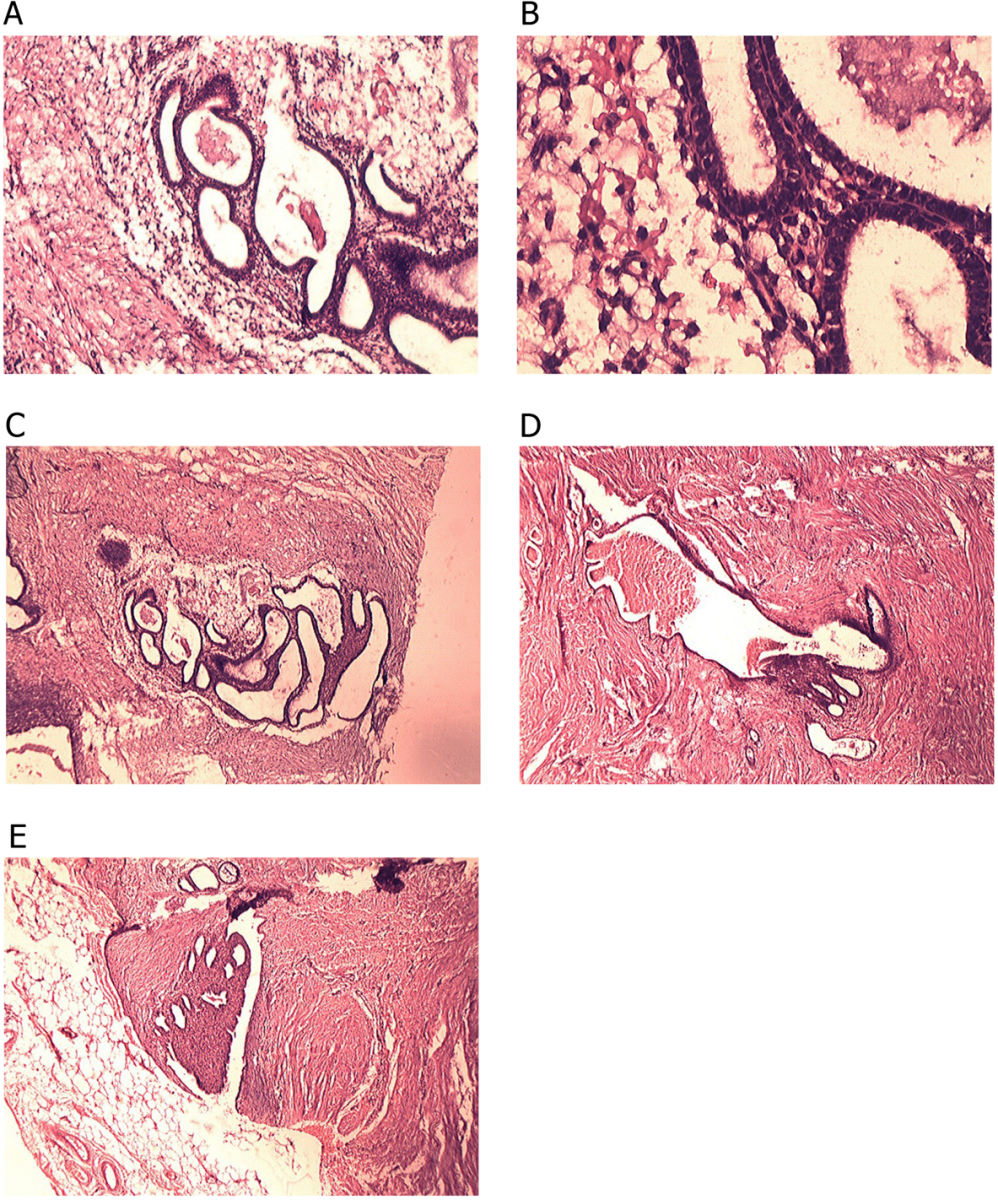
**Histopathological characteristics of endometriosis in different tissue types. A**: Endometrial strauma and glands in fatty connective tissue (X10). **B**: Endometrial strauma and glands in fatty connective tissue (X40). **C**: Endometrial strauma and glands in fibro-connective tissue (X10). **D**: Endometrial strauma and glands in muscular tissue (X10). **E**: Endometrial strauma and glands in muscular tissue adjacent to fatty layer (X10).

Histological confirmation of endometrioma is based on detecting at least two of the three following features: endometrial stroma, endometrial like glands and hemosiderin pigment [10]. After histopathological confirmation (Figure 1), the data of 30 patients who underwent AWE surgery were entered in our study. All the patients had at least two features of endometrioma. Table 1 summarizes the characteristics of the patients with abdominal wall endometriosis. The age of the patients ranged from 23 to 67 years. The mean age of the patients was 32.5 ± 8.24 years. Their number of parities ranged from one to four. We found a previous history of caesarean section in all of the patients. Eleven patients (36.6%) had a single caesarean section. Fourteen patients (46.7%) had two caesarean sections and five (16.7%) had three caesarean sections. Eight patients (26.7%) had surgeries other than caesarean section. These surgeries include one myomectomy, three hysterectomies, one tubalectomy, and three appendectomies. All patients had a notable mass as a presenting symptom. Other than the mass, 24 patients (80%) experienced pain, among whom 14 patients (46.7%) experienced cyclic pain. Two patients (6.7%) had dyspareunia, three (10%) had dysmenorrhea, and one (3.3%) had bleeding as presenting symptoms. Four patients did not have any symptoms and AWE was discovered during the caesarean sections. The mean duration of symptoms was 12.7 ± 10.44 months, ranging from three to forty-five months. The mean time between the previous surgery and diagnosis of AWE was 30.5 ± 12.65 months. In 28 patients (93.3%), the ultrasonography was used to diagnose AWE, computed tomography (CT) scan and magnetic resonance imaging (MRI) were used in five (16.6%) and two (6.6%) patients, respectively. Twenty-seven patients (90%) had a single mass, whereas, three (10%) had two masses at the time of diagnosis. In nineteen patients (63.3%), the initial diagnosis was AWE, whereas abdominal wall tumour was diagnosed in four patients (13.3%), incisional hernia in three (10%) cases, suture granuloma in two cases (6.7%), and inguinal hernia and desmoid tumour each in one case (3.3%). The location of the masses was in or under the scar of the previous surgery in 21 patients (70%) (in three cases the mass was located in the scar), hypochondrium away from the scar and umbilicus each in four cases (13.3%), and groin region in one case (3.3%). The mean diameter of the masses was 2.59 ± 1.21 cm, ranging between 1 cm and 6 cm. Nine cases (30%) had a mass in the fat tissue, fourteen (46.7%) in muscle tissue, eleven (36.7%) in the fascia of the abdomen muscles, ten (33.3%) in the omentum, and one (3.3%) in the dermis. Hematoma and necrosis were observed in one (3.3%) and two patients (6.7%), respectively. No case of malignancy was reported among our study patients. All the patients underwent follow-up tests and one patient was diagnosed with a recurred mass after six months, which was removed with clear margins.

**Table 1:** **Characteristics of the patients with abdominal wall endometriosis**

	**n**	**%**	**Range**	**Mean +/- SD**
**Age**			23-67	32.5 ± 8.24
**Parity**			0-4	2
One CS	11	36.6		
Two CS	14	46.7		
Three CS	5	16.7		
**Surgeries other than CS**				
Myomectomy	1	3.3		
Hysterectomy	3	10		
Tubalectomy	1	3.3		
Appendectomy	3	10		
**Presenting symptom**				
Mass	30	100		
Pain (noncyclic)	10	33.3		
Pain (cyclic)	14	46.7		
Dyspareunia	2	6.7		
Dysmenorrhea	3	10		
Bleeding	1	3.3		
**Duration of symptoms (months)**			3-45	12.77 ± 10.44
**Asymptomatic period (months)**			2-53	30.5 ± 12.65
**Diagnosing tool**				
Ultrasonography	28	93.3		
CT	5	16.6		
MRI	2	6.6		
Single mass	27	90		
Two masses	3	10		
**Accurate diagnosis**	19	63.3		
**Wrong diagnosis**	11	36.7		
**Location of the mass**				
Under the scar	18	60		
In the scar	3	10		
Far from the scar	9	30		
**Diameter of the mass (cm)**			1-6	2.59 ± 1.21

CS: Caesarean Section.

## Discussion

Caesarean section and hysterectomy are considered to be commonly associated with AWE, and the surgical scar of the caesarean section is the most common site for extra pelvic endometriosis known as caesarean scar endometriosis with the incidence of 0.07%–0.47% [4]. In the current study, all the patients with AWE had a prior history of caesarean section and some patients had another gynaecological surgery such as hysterectomy besides caesarean section. In our study, the majority of patients had one or two caesarean sections.

There are two theories that are more accepted for the cause of AWE: transport theory and metaplasia theory. The transport theory explains that direct inoculation or transport of the endometrial tissue into the surgical scars or adjacent tissues during the surgery is responsible for AWE. In metaplasia theory, primitive pleuropotential mesenchymal cells that have gone under differentiation metaplasia are said to cause AWE. Alternative theories like lymphatic or haematogenic dissemination and cell immunity change theory are among the other proposed theories [8,10]. The fact that all the patients of our study had undergone a cesarean section, and some had other gynecological surgeries may be in favor of transport theory. This theory might explain that endometrial tissues can be iatrogenically transported into the abdominal wall or into the scars of the surgery. Although the presence of endometrial tissues in the abdominal wall can be explained with this theory, some other features of endometriosis, like why the endometrial tissues can survive and proliferate in the new sites cannot be explained with the transport theory.

Mass sensation, pain, dysmenorrhea and bleeding are among the symptoms of endometriosis. Although the cycle symptoms may be pathognomonic of endometriosis, the symptoms can be non-cyclic, too. Endometriosis can be categorized as a steroid-dependant disease [13]. It is known that oestrogens can trigger the development of endometriotic lesions. Endometrial cells can be stimulated with oestrogen and proliferate until they become symptomatic. The oestrogen can be further produced in the endometrial lesions by aromatase activity [14]. All the patients in our study had symptoms of abdominal mass sensation. Additionally, 46.7% of them experienced cyclic pain and 33.3% experienced noncyclic pain. Dysmenorrhea and bleeding were the presenting symptoms in 10% and 3.3% of the patients, respectively. Ultrasonography is mentioned as the most frequently used technique for diagnosing AWE and we used this diagnostic tool in 93.3% of the patients. CT scan and MRI were the least used methods.

Not all of the endometriosis patients had cyclic symptoms. The diagnosis of this disease might be challenging and several differential diagnoses might exist for a mass in the abdominal wall, including desmoid tumour, fibrosis, suture granuloma, fat necrosis, nodular fasciitis and primary or metastatic malignancies lesions [15]. Although the history of previous gynaecological surgeries might be helpful and leading us to AWE, the definite diagnosis of endometriosis might be possible only after the surgery and removal of the mass and pathological evaluations. Using of fine needle aspiration cytology (FNAC) can be helpful before the surgery and can be used for determining all three criterion of endometriosis [15], but it was not performed in our study.

The presence of AWE in the surgery scar is quite infrequent. The series of one, five, and six scar endometriosis had been reported earlier [11,16,17]. We only found ten percent of the masses of the patients (3 masses) were located within the scars of the previous caesarean sections.

Although the risk of malignancy transformation of endometriosis is not known and less than 1% of endometriosis patients are reported to develop associated neoplasms, the most common type is clear-cell carcinoma. This neoplasm has 5-year survival of 80% [8]. Although there are some reports of clear-cell carcinoma associated with AWE [18], there was none in our study.

## Conclusion

In conclusion, it is worth saying that although abdominal wall endometriosis is a rare disorder, it can occur after gynaecological surgeries and caesarean sections especially near or within the scars. As caesarean section rates are becoming more frequent, and there is a high prevalence of caesarean section among women with AWE, we may encounter higher rates of this uncommon entity in the future.
